# A case report of atypical chronic myeloid leukemia with complete hematological and major molecular response to Venetoclax/Azacitidine treatment

**DOI:** 10.3389/fonc.2024.1327834

**Published:** 2024-03-25

**Authors:** Hongxia Chen, Ning Wang, Yin Li, Xiaohong Xie, Yi Yang

**Affiliations:** ^1^Department of Hematology, Chongqing University Three Gorges Hospital, Chongqing, China; ^2^School of Medicine, Chongqing University, Chongqing, China; ^3^Department of Medicine, Northwestern University, Chicago, IL, United States

**Keywords:** atypical chronic myeloid leukemia, Venetoclax, Azacitidine, combination therapy, allo-transplantation

## Abstract

Atypical Chronic Myeloid Leukemia (aCML), a myeloproliferative neoplasm with poor prognosis, was reclassified as aCML by the ICC classification, and as MDS/MPN with neutrophilia by the WHO 2022 classification. Due to the heterogeneity of its clinical features and the lack of unique biomarkers, as well as limited treatment options, aCML currently lacks a standardized treatment protocol. In this case report, we reviewed a young man diagnosed with aCML who achieved complete clinical and hematologic remission subsequent to receiving a therapeutic regimen combining Venetoclax and Azacitidine.

## Introduction

Atypical Chronic Myeloid Leukemia (aCML) is a myeloproliferative neoplasm with poor prognosis due to its significant risk of progressing into acute myeloid leukemia(AML) (1). It was reclassified as aCML by the ICC classification, and as MDS/MPN with neutrophilia by the WHO 2022 classification. aCML is characterized by marked leukocytosis/granulocytosis like CML, yet it lacks discernible evidence of the classic t(9;22) BCR-ABL1 translocation as determined through cytogenetic studies, polymerase chain reaction (PCR), or fluorescence situ hybridization (FISH) (2, 3). Due to the heterogeneity of its clinical features and the lack of unique biomarkers, as well as limited treatment options, aCML currently lacks a standardized treatment protocol (4). In this case report, we reviewed a young man diagnosed with aCML who achieved a complete clinical and hematologic remission subsequent to receiving a therapeutic regimen combining Venetoclax and Azacitidine.

## Case report

**Figure 1:**
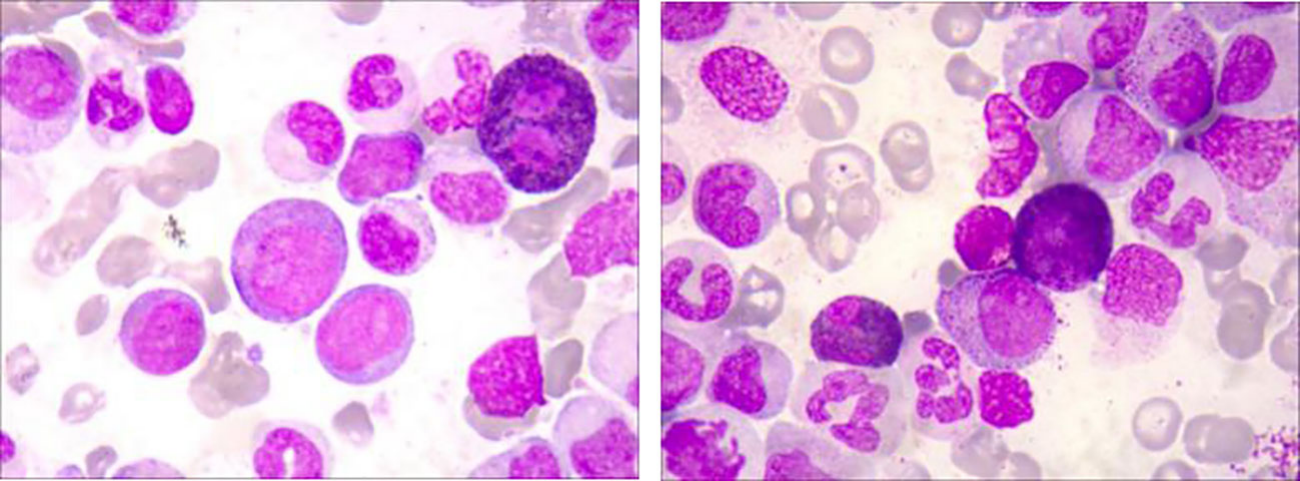
Bone marrow (BM) smears of the patient at the diagnosis.

A 41-year-old male with an unremarkable medical history had been suffering from abdominal bloating for one month before presenting to our Hematology department. The complete blood count showed a high white blood cell (WBC) count of 210.74×10^9^/L, a low hemoglobin (Hb) level of 68g/L, and a low platelet (PLT) count of 17 ×10^9/L. A manual differential revealed distinct abnormalities in the white blood cell distribution: 95% neutrophils (11% segmented neutrophils, 12% band neutrophils, 27% metamyelocytes, 44% myelocytes, and 1% promyelocytes), 1% monocytes, 2% blast cells and 2% lymphocytes. Physical examination indicated hepatosplenomegaly. A bone marrow (BM) examination and trephine biopsy were performed and revealed hypercellularity with marked myeloid expansion with primary + promyelocytic granulocytes accounting for 7.5% (**Figure 1**). These granulocytes mainly predominantly exhibited various developmental stages, including middle, late, and rod-shaped forms, with an unbalanced nuclear morphology and decreased or absent cytoplasmic granules. Flow Cytometry analysis uncovered an abnormal myeloid protocell phenotype characterized by CD34+CD117+CD38dimHLA-DRdimCD13+CD33+CD11B-CD15-CD64-CD56-CD7-, accounting for 1.63% of nucleated cells. Karyotype was normal and an initial molecular analysis targeting BCR/ABL1 rearrangement, as well as mutations of CSF3R, JAK2V617F, CARL, and MPL, did not reveal any genetic abnormalities. As a next step, the diagnostic bone marrow sample underwent next generation sequencing (NGS) analysis utilizing the Myeloid Proliferative Neoplasm Sequencing Panel (Illumina), covering 77 genes that are known to be frequently mutated in myeloid malignancies, which unveiled pathogenic variants in ASXL (p.Y591*, VAF 47.9%), CEBPA(p.L78Wfs*82, VAF 46%), vKRAS(p.A146T, VAF 21.1%), NRAS(p.G12R, VAF 21.4%), and STAG2 (p.L351Tfs*6, VAF 73.9%) (Clinic features were showing in **Table 1**).

**Table 1:** Characteristics at Diagnosis and Outcome of the Clinical Cases of aCML.

Clinic Featrures	
Age(Years)	41
WBC	×109/L
Diferential (PB)	hypogranulated neutrophils
	band-cells 12%
	eosinophils 0.5%
	basophils -
	monocytes 1%
	lymphocytes 1%
	myeloblasts 2%
	promyelocytes 1%
	metamyelocytes 27%
	myelocites 44 %
Hb	68
Plts	17
BM blasts	0.02
Screening for mutations in JAK2,CALR and MPL	negative
BCR/ABL	negative
Gene mutations identified by NGS	ASXL (p.Y591*, VAF 47.9%), CEBPA(p.L78Wfs*82, VAF 46%),vKRAS(p.A146T, VAF 21.1%), NRAS(p.G12R, VAF 21.4%), and STAG2 (p.L351Tfs*6, VAF 73.9%).
Karyotype	46, XY
Treatment	Azacitidine+Venetoclax
Follow-up time (months after diagnosis)	8 months
Status at last follow up	Alive in complete remission

**Figure 2:**
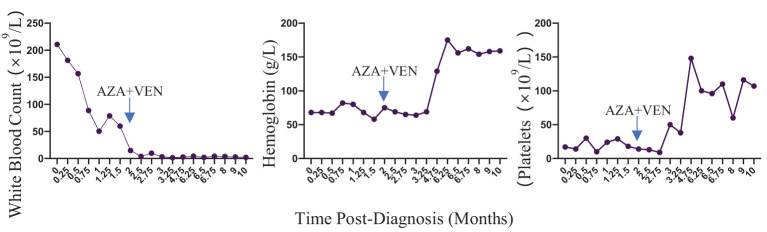
Blood counts of the patient during the treatment.

The diagnosis of aCML was made in concordance with the 2022 WHO diagnostic criteria for aCML. Due to the absence of a standardized medical protocol for aCML, a thorough evaluation for stem cell transplantation was recommended, but ultimately declined by the patient. He underwent leukocyte removal to alleviate leukocytosis, reduce blood viscosity and improve microcirculation. He was initially started on both hydroxyurea and imatinib, with concurrent evaluation for imatinib, given the strikingly similar clinical presentation to typical CML. However, imatinib administration was stopped subsequent to a negative result for the BCR/ABL1 rearrangement. Subsequently he received 3 cycles of a 5-day regimen consisting of cytarabine and azacytidine. Unfortunately, the changes of peripheral blood, bone marrow and hepatosplenomegaly did not obviously improve compared with the beginning of the disease, and the cervical lymph nodes were enlarged, indicating suboptimal disease control. Due to the patient’s worsening blood counts and limited alternative treatment options, a therapeutic approach combining venetoclax(400mg daily for 21days) and azacytidine(100mg daily for 7days) was initiated. He had rapid clinical improvements of blood counts (**Figure 2**), bone marrow, cervical lymph nodes enlargement and hepatosplenomegaly after 1 month of the combination therapy. Then he continued to receive the same combination therapy once a month for. The bone marrow smear showed hyperplasia was active, no primary or promyelocytic granulocytes were seen and the immune residue showed negative after 8 months of the treatment. He continued to maintain complete clinic remission at the latest follow-up after 10 months of the treatment.

## Discussion

The age of onset for aCML predominantly occurs around 60 years of age, with approximately half of the patients exhibiting concurren hepatosplenomegaly, increased WBC count, anemia and thrombocytopenia. Due to the low incidence, specific molecular abnormalities associated with aCML have yet to be identified to date, leading to significant challenges in the clinical diagnosis of this condition. Our patient was diagnosed with aCML defined by his BCR-ABL1(-) status in the setting of marked leukocytosis/granulocytosis and prominent dysgranulopoiesis, and granulocytic dysplasia, according to the 2022 WHO diagnostic criteria for aCML.

aCML is a rare disease, and standard treatment approaches are lacking both domestically and internationally. Patients should be treated promptly when they manifest progressive leukocytosis, anemia, thrombocytopenia, spleen enlargement, or other disease-associated symptoms (2, 5). Allogeneic Hematopoietic Stem Cell Transplantation (allo-HSCT) is recommended as a preferred option for younger patients with a suitable donor; however, the optimal timing of transplantation remains controversial (6). Given that aCML exhibits numerous resemblances to other chronic myeloid disease, its treatment strategy can be referred to Myelodysplastic Syndromes (MDS) or Myeloproliferative Neoplasms (MPN). This can involve the use of agents like hydroxyurea or interferon to mitigate tumor burden and alleviate splenomegaly-related symptoms. Regrettably, these interventions tend to yield suboptimal results, particularly in advanced stages of the disease (2). Based on the good efficacy of demethylated drugs such as decitabine or azacitidine in Chronic Myelomonocytic Leukemia (CMML), some studies have tried to use these drugs in aCML treatment (7). So far, it has been reported that the complete response (CR) rate of treating aCML with decitabine alone can reach 40%, but the long-term survival benefit still needs to be confirmed by larger sample analyses and longer follow-up observations (8). Notable elevations in leukocyte count, combined with anemia, thrombocytopenia and splenomegaly, prompted the necessity for treatment, but the economic conditions for transplantation were not available, and the effectiveness of adding hydroxyurea to reduce leukocytes proved limited. In order to minigrate disease progression and improve patient survival, azacitidine induction therapy was administered.

With the continuous development of molecular diagnostic technologies, there has been an increasing detection of mutated genes in aCML, leading to a deeper understanding of this condition, which, in turn, holds significant implications for its treatment (9). Some clinical reports have shown that Ruxolitinib can improve the prognosis of aCML patients (10). However, in the case of this patient who presented with anemia and thrombocytopenia and exhibited no mutations in the CSF3R or JAK2 genes, the use of Ruxolitinib was not considered. B-cell lymphoma-2 (BCL-2), a key regulator of the mitochondrial apoptotic pathway, is overexpressed in many hematological malignancies, especially within leukemia stem cells (11). Overexpression of BCL-2 has been shown to reduce the survival rate of AML cells and is associated with chemotherapy resistance (12). Venetoclax is currently the only approved BCL-2 inhibitor. As a BH3 analogue, venetoclax can simulate the selective binding of BH3-only pro-apoptotic protein to BH3 domain of anti-apoptotic protein in BCL-2 family, effectively inhibit BCL-2, and affect the permeability of mitochondrial outer membrane, leading to the rapid initiation of apoptosis (12, 13). *In vitro* studies have illuminated that venetoclax can increase the sensitivity of AML cells to demethylation drugs (14). Furthermore, demethylation drugs can reduce the level of anti-apoptotic protein MCL-1 and delay the emergence of venetoclax resistance (15). Therefore, the patient was treated with azacytidine combined with the BCL-2 targeting drug venetoclax, and the patient had obtained CR, and minimal residual disease (MRD) turned negative early to the follow-up cut-off point, the survival time of the patient was 10 months, and the prognosis was significantly improved. Even though this therapeutic regimen combining Venetoclax and Azacitidine could promote complete clinical and hematologic remission for, but the long-term benefit is unknown, so allo-transplantation should also be initialed as soon as possible to achieve long-term survival.

## Conclusion

The diagnosis of aCML is difficult, and only morphological changes may cause certain missed diagnosis in clinic. The relentless progress in molecular biology technology has led to the continual detection of MDS/MPN-related molecular markers, thereby enriching our comprehension of aCML. The patient, aged 41, had no transplantation conditions. Remarkable clinical efficacy was achieved after treatment with azacitidine combined with venetoclax, with good hematological and molecular responses and high safety. However, the long-term clinical outcome still needs to be further followed up and evaluated.

## Data availability statement

The datasets presented in this study can be found in online repositories. The names of the repository/repositories and accession number(s) can be found in the article/supplementary material.

## Ethics statement

The case report was approved by the medical research center. The patient has provided written informed consent for the publication of this case report.

## Author contributions

HC: Writing – original draft. YL: Writing – original draft. YY: Writing – review & editing. XX: Writing – original draft. NW: Writing – review & editing, Project administration.
